# Sex-related outcomes after percutaneous coronary intervention of in-stent restenosis

**DOI:** 10.1007/s12928-025-01092-y

**Published:** 2025-02-03

**Authors:** Constantin Kuna, Christian Bradaric, Mira Schroeter, Antonia Presch, Felix Voll, Sebastian Kufner, Tareq Ibrahim, Heribert Schunkert, Karl-Ludwig Laugwitz, Salvatore Cassese, Adnan Kastrati, Jens Wiebe

**Affiliations:** 1 Deutsches Herzzentrum München, Department of Cardiology, Technische Universität München, Lazarettstr. 36, 80636 Munich, Germany; 2 DZHK (German Centre for Cardiovascular Research), Partner Site Munich Heart Alliance, Munich, Germany; 3 Clinic and Policlinic Internal Medicine I (Cardiology and Angiology), Klinikum rechts der Isar, Technische Universität München, Munich, Germany

**Keywords:** Sex, Drug-coated balloon, Drug-eluting stent, In-stent restenosis, Percutaneous coronary intervention

## Abstract

Limited data are available for sex-related long-term outcomes of patients treated for coronary drug-eluting stent (DES) restenosis. The aim of this observational, retrospective analysis was to close this lack of evidence. Between January 2007 and February 2021, a total of 3511 patients with 5497 in-stent restenosis (ISR) lesions were treated at two large-volume centers in Munich, Germany, of which 763 (21.7%) were female. Endpoints of interest were all-cause mortality and rates of repeat revascularization. Outcomes are presented as Kaplan–Meier event rates. Univariate and multivariate analyses were performed. Female patients were older (72.1 ± 10.4 versus 68.4 ± 10.4 years, *p* < 0.001) and suffered more often from diabetes (38.8% versus 34.4%, *p* = 0.029). There was no statistical difference regarding the clinical presentation and interventional treatment strategy. After 10 years, the risk of all-cause mortality was higher in female patients [hazard ratio (HR) 1.18 (1.02–1.35); *p* = 0.022]; however, after adjustment for age, the risk did not differ significantly between both sexes [adjusted HR 0.96 (0.83–1.11); *p* = 0.6]. Regarding target vessel revascularization (TVR) and non-target vessel revascularization (NTVR), the risk was lower in female patients [HR 0.73 (0.61–0.87); *p* < 0.001 and HR 0.74 (0.64–0.86); *p* < 0.001] even after age adjustment. No statistical differences were found regarding the risk of target lesion revascularization, target vessel myocardial infarction and stent thrombosis. In the long term, all-cause mortality after percutaneous coronary intervention of DES-ISR did not differ between both sexes after age adjustment. The risk of TVR and NTVR was lower in female patients even after age adjustment.

**Graphical abstract:**

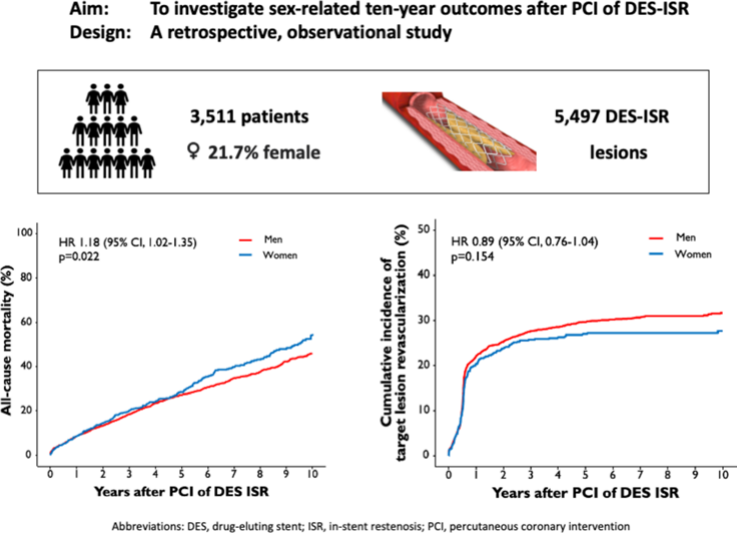

## Introduction

In both female and male patients, ischemic heart disease constitutes the most common cause of cardiovascular death in European Society of Cardiology (ESC) member countries [1]. Over time, methodological advances in percutaneous coronary intervention (PCI) have improved outcomes in patients with obstructive coronary artery disease (CAD) [2]. Despite the implementation of drug-eluting stents (DES), a considerable number of patients still develop in-stent restenosis (ISR) [3] which represents the most common cause of treatment failure after PCI and goes along with increased mortality rates [4].

Previous data reported inherent biological factors which contribute to sex differences in cardiovascular disease [5] and which are difficult to adjust [6]. Female patients develop cardiovascular disease an average of 10 years later than male patients [7]. Moreover, an older age on admission and non-typical angina pectoris decelerate diagnosis and revascularization in female compared to male patients [8]. Data evaluating sex-related outcomes after PCI yielded conflicting results [9–13] with a trend towards higher risk of adverse outcomes in female patients [14].

However, data comparing clinical outcomes between female and male patients after PCI of DES-ISR is scarce as most sex-specific data refer to treatment of native coronary artery stenosis. Especially since the underlying mechanism is different, a generalization of the available sex-specific PCI data for all settings is difficult and a separate analysis of patients with ISR appears necessary. Considering the general lack of long-term data after PCI, information on sex-specific long-term outcomes after PCI of DES-ISR are even more desirable. Thus, the aim of this analysis is to close this gap of knowledge by providing sex-related long-term data after PCI of DES-ISR.

## Methods

### Study design and patient selection

The ISAR-DESIRE registry is an observational, retrospective study of patients who underwent PCI for the treatment of DES-ISR between January 2007 and February 2021 at the Deutsches Herzzentrum München, Munich, Germany and the 1. Med. Klinik, Klinikum rechts der Isar, Technical University, Munich, Germany. The present analysis focuses on the development of ISR in dependence of male vs. female sex. The study was approved by the local ethics board of the Technical University Munich, Germany and adheres to the Declaration of Helsinki.

### Interventional procedure and medications

PCI of DES-ISR was performed according to standard clinical practice. All patients were treated with either balloon angioplasty (BA) (including plain balloon angioplasty and drug-coated balloon [DCB] angioplasty) or implantation of DES. The decision regarding the individual therapy strategy was determined by the treating physician. According to local standards, pre-dilatation was recommended for all patients undergoing PCI for ISR, the use of scoring or debulking devices was left to the operators’ discretion, as well as the decision to perform post-dilatation after stenting. During the procedure, patients received intravenous and body weight-adjusted intraarterial or intravenous heparin or bivalirudin. After the intervention, all patients received aspirin indefinitely and a P2Y12 inhibitor (clopidogrel, prasugrel, or ticagrelor) according to clinical presentation and recommendations at the time of PCI.

### Data collection

Personnel of the clinical data management center (ISAResearch-Center, Munich, Germany) collected relevant data on source-documented hospital chart reviews. These data included information regarding clinical and lesion-related parameters at the time of initial and repetitive PCIs. The required data for this study were entered into an anonymized study database. Angiographic parameters of interest included the type of intervention (BA vs. DES), balloon diameter and maximum balloon pressure, total stented length, and stent diameter as well as thrombolysis in myocardial infarction (TIMI) grade flow pre- and post-PCI.

### Endpoints

Endpoints of interest regarding this sex-related analysis are all-cause mortality, target lesion revascularization (TLR), target vessel revascularization (TVR), target vessel myocardial infarction (TVMI), non-target vessel revascularization (NTVR), non-target vessel myocardial infarction (NTVMI) and stent thrombosis (ST). TVR is defined as revascularization in the target vessel outside the target lesion. Event rates are presented as Kaplan–Meier 10-year event rates for all-cause mortality and cumulative 10-year incidences after accounting for the competing risk of death for the other endpoints. The definition of myocardial infarction (MI) used in this analysis is based on the Third Universal Definition of Myocardial Infarction [15]. The definitions of ISR, ST, TLR and NTVR used in this trial are adapted from the Academic Research Consortium [16, 17].

### Statistics

Baseline descriptive parameters are presented as mean ± standard deviation for continuous data and numbers with percentages for categorical data. Survival analyses were performed according to Kaplan–Meier method and the treatment of the first DES-ISR was defined as time zero. Univariate analyses considering clinical and lesion-related variables were performed. Multivariable analyses were added for selected parameters associated with clinical outcomes. Adjusted analyses were performed for the following variables: sex, age, diabetes mellitus, multivessel CAD, prior MI and prior CABG. Unadjusted and adjusted hazard ratios (HR) and 95% confidence intervals were calculated with the use of Cox proportional models. A special analysis was performed for recurrent ISR without censoring after the first event. An extended Cox model (Andersen–Gill model) was applied in this case that accounted for the competing risk of death. Robust sandwich estimators for variance of regression coefficients were used to account for within-subject correlation. A 2-tailed *p* value of less than 0.05 was considered statistically significant. Statistical software R (Version 4.0, R Foundation for Statistical Computing, Vienna, Austria) was used for all analyses.

## Results

### Baseline and procedural characteristics

A total of 3511 patients underwent PCI due to DES-ISR. 21.7% (763 patients) were female and significantly older than male patients (72.1 ± 10.4 years vs. 68.4 ± 10.4 years; *p* < 0.001). Female patients suffered from diabetes in 38.8%, male patients in 34.4% (*p* = 0.029). There were no statistically relevant differences found regarding other cardiovascular risk factors. Female patients less often presented with coronary multivessel disease than males (84.1% vs. 91.2%; *p* < 0.001). Female patients were less likely to have prior myocardial infarction (41.5% vs. 46.1%; *p* = 0.029) and prior coronary artery bypass graft (CABG) surgery (10.6% vs 14.8%; *p* = 0.004) than male patients. In both groups, most patients were admitted due to stable CAD. Further clinical baseline characteristics are presented in Table 1.

**Table 1:** Clinical baseline characteristics

	Female patients (*n* = 763)	Male patients (*n* = 2748)	*p* value
Age (in years)	72.1 ± 10.4	68.4 ± 10.4	< 0.001
BMI (in kg/m^2^)	27.3 ± 5.7 (*n* = 739)	27.6 ± 4.1 (*n* = 2692)	0.128
Diabetes mellitus	296 (38.8)	946 (34.4)	0.029
Insulin dependent	123 (16.1)	348 (12.7)	0.016
Arterial hypertension	717 (94.0)	2551 (92.8)	0.309
Hypercholesterolemia	555 (72.7)	2032 (73.9)	0.534
Current smoker	98 (12.8)	422 (15.4)	0.095
CAD			< 0.001
1-vessel CAD	121 (15.9)	243 (8.8)	
2-vessel CAD	174 (22.8)	533 (19.4)	
3-vessel CAD	468 (61.3)	1972 (71.8)	
Multivessel CAD	642 (84.1)	2505 (91.2)	< 0.001
Ejection fraction (in %)	54.8 ± 10.7 (*n* = 481)	52.1 ± 11.2 (*n* = 1680)	< 0.001
Clinical presentation			0.528
Stable angina	523 (68.5)	1959 (71.3)	
Unstable angina	118 (15.5)	389 (14.2)	
NSTEMI	99 (13.0)	328 (11.9)	
STEMI	23 (3.0)	72 (2.6)	
Prior CABG	81 (10.6)	407 (14.8)	0.004
Prior MI	317 (41.5)	1266 (46.1)	0.029
Creatinine on admission (in mg/dl)	1.0 ± 0.4	1.1 ± 0.4	< 0.001
CRP on admission (in mg/l)	5.7 ± 15.9	4.9 ± 15.0	0.216
ASA on discharge	727 (95.3)	2,670 (97.2)	0.013
P2Y12 inhibitor on discharge	712 (93.3)	2,619 (95.3)	0.035
Statine on discharge	679 (89.0)	2,527 (92.0)	0.012

Data shown as means ± standard deviation or number (percentage)

ASA, acetylsalicylic acid; BMI, body mass index; CABG, coronary artery bypass graft; CAD, coronary artery disease; CRP, C-reactive protein; MI, myocardial infarction; NSTEMI, non-ST-elevation myocardial infarction; STEMI, ST-elevation myocardial infarction

A total of 5497 ISR lesions were treated. In female patients, DES-ISR was treated with another DES implantation in 53.0% and with BA in 47.0%, in male patients with DES implantation in 53.5% and with BA in 46.5% (*p* = 0.818). In the BA group, 40.0% (211/528 patients) of the female and 37.9% (772/2,035 patients) of the male patients were treated with a DCB. Mean maximum balloon pressure was 15.0 atm (13.0–18.0 atm) in female and 16.0 atm (13.0–18.0) in male patients (*p* < 0.001). Mean maximum stent diameter was 3.0 mm (2.75–3.5 mm) in female (*p* < 0.001) and 3.0 mm (3.0–3.5 mm) in male patients, and mean stented length was 23.0 mm (18.0–31.5 mm) in female and 23.0 mm (18.0–30.0 mm) in male patients (*p* = 0.862). A summary of procedure-related characteristics is shown in Table 2.

**Table 2:** Procedural characteristics

	Lesions in female patients (*n* = 1124)	Lesions in male patients (*n* = 4373)	*p* value
Type of intervention			0.818
Balloon angioplasty	528 (47.0)	2035 (46.5)	
Stenting	596 (53.0)	2338 (53.5)	
Drug-coated balloon	211 (18.8)	772 (17.7)	0.407
Cutting balloon	33 (2.9)	110 (2.5)	0.493
Bifurcation lesion	354 (31.6)	1459 (33.6)	0.211
Ostial lesion	369 (32.9)	1314 (30.2)	0.095
Max. balloon diameter (mm)	3.0 (3.0; 3.5)	3.0 (3.0; 3.5)	0.019
Max. balloon pressure (atm)	15.0 (13.0; 18.0)	16.0 (13.0; 18.0)	0.042
Max. stent diameter (mm)	3.0 (2.8; 3.5) (*n* = 588)	3.0 (3.0; 3.5) (*n* = 2311)	< 0.001
Total stented length (mm)	23.0 (18.0; 31.5) (*n* = 588)	23.0 (18.0; 30.0) (*n* = 2311)	0.862
TIMI grade flow pre-PCI			0.963
0	88 (7.8)	333 (7.7)	
1	20 (1.8)	86 (2.0)	
2	77 (6.9)	289 (6.6)	
3	939 (83.5)	3649 (83.8)	
TIMI grade flow post-PCI			0.475
0	18 (1.6)	81 (1.9)	
1	6 (0.5)	12 (0.3)	
2	20 (1.8)	90 (2.1)	
3	1079 (96.1)	4172 (95.8)	

Data shown as number (percentage) or absolute numbers (95% confidence interval)

PCI, percutaneous coronary intervention; TIMI, thrombolysis in myocardial infarction

### Clinical outcomes

**Fig. 1:**
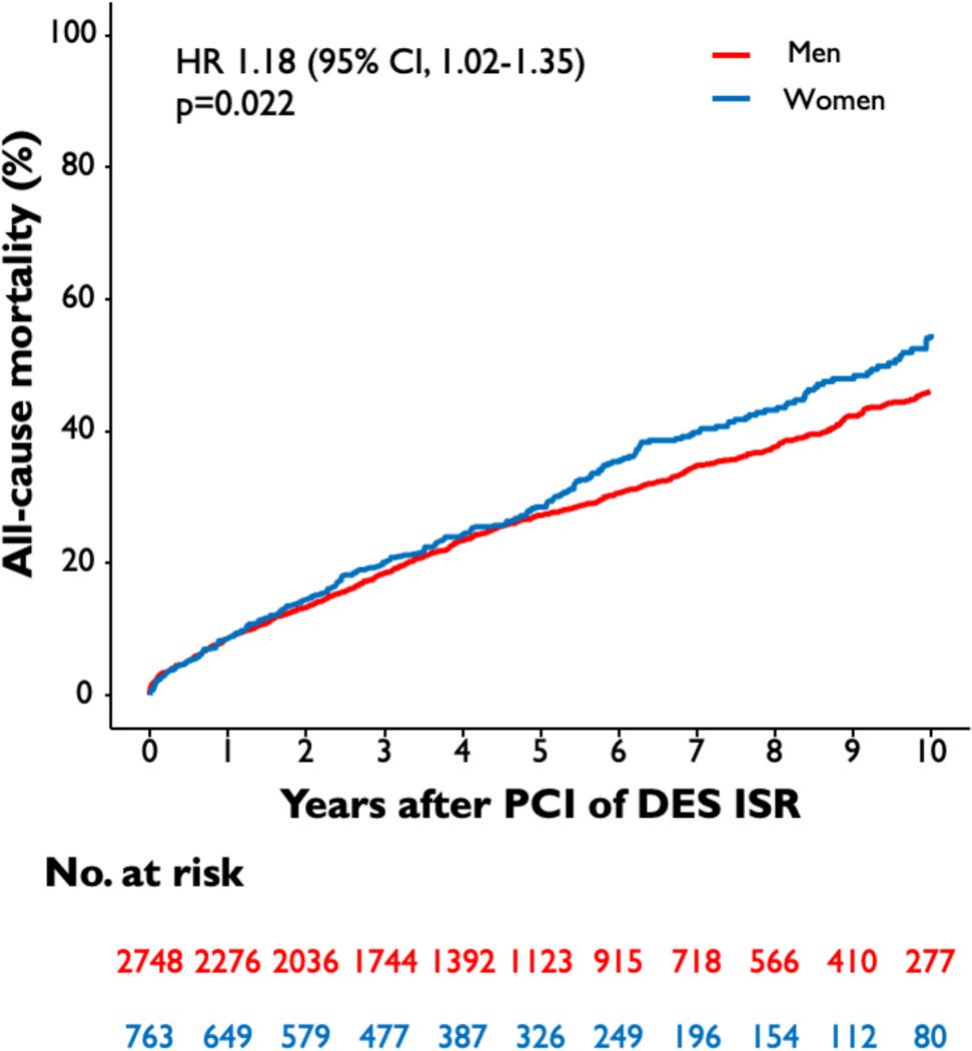
Sex-related 10-year incidence of all-cause mortality. The figure demonstrates the cumulative incidence function curve and HR with accompanying 95% CI

**Fig. 2:**
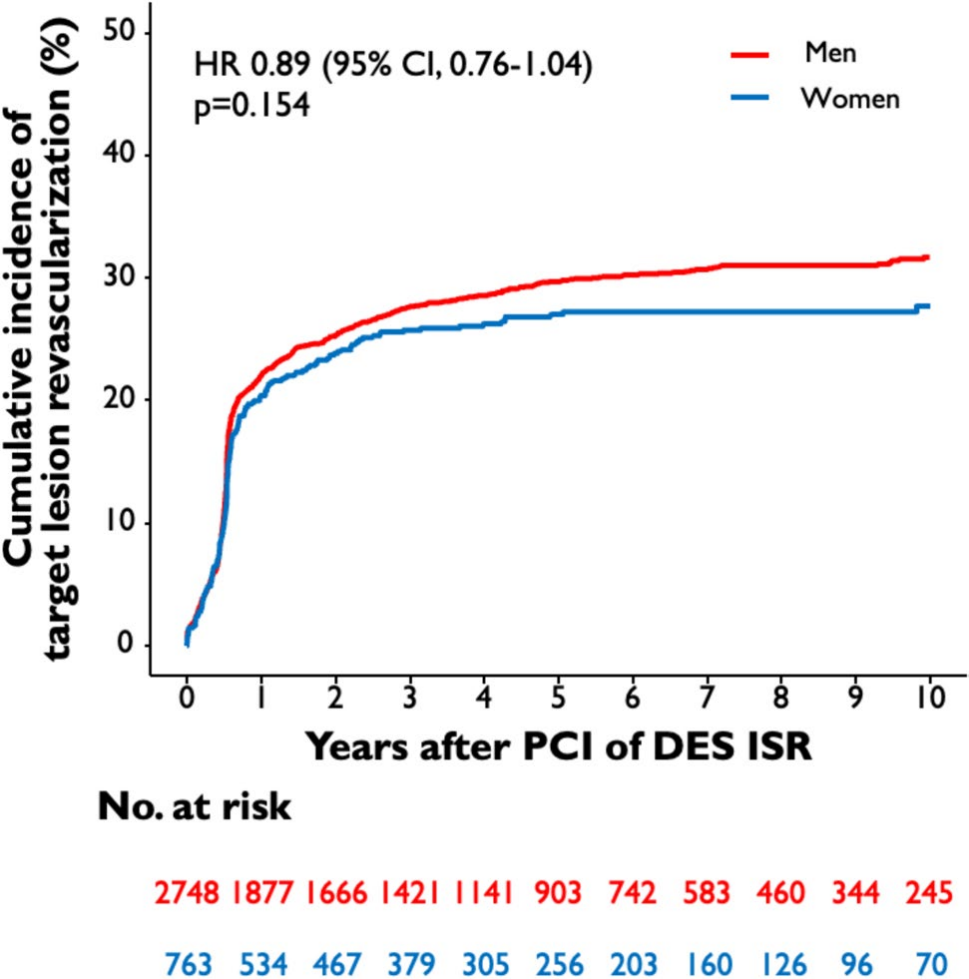
Sex-related 10-year cumulative incidence of TLR. The figures demonstrate the cumulative incidence function curves and HR with accompanying 95% CI

**Fig. 3:**
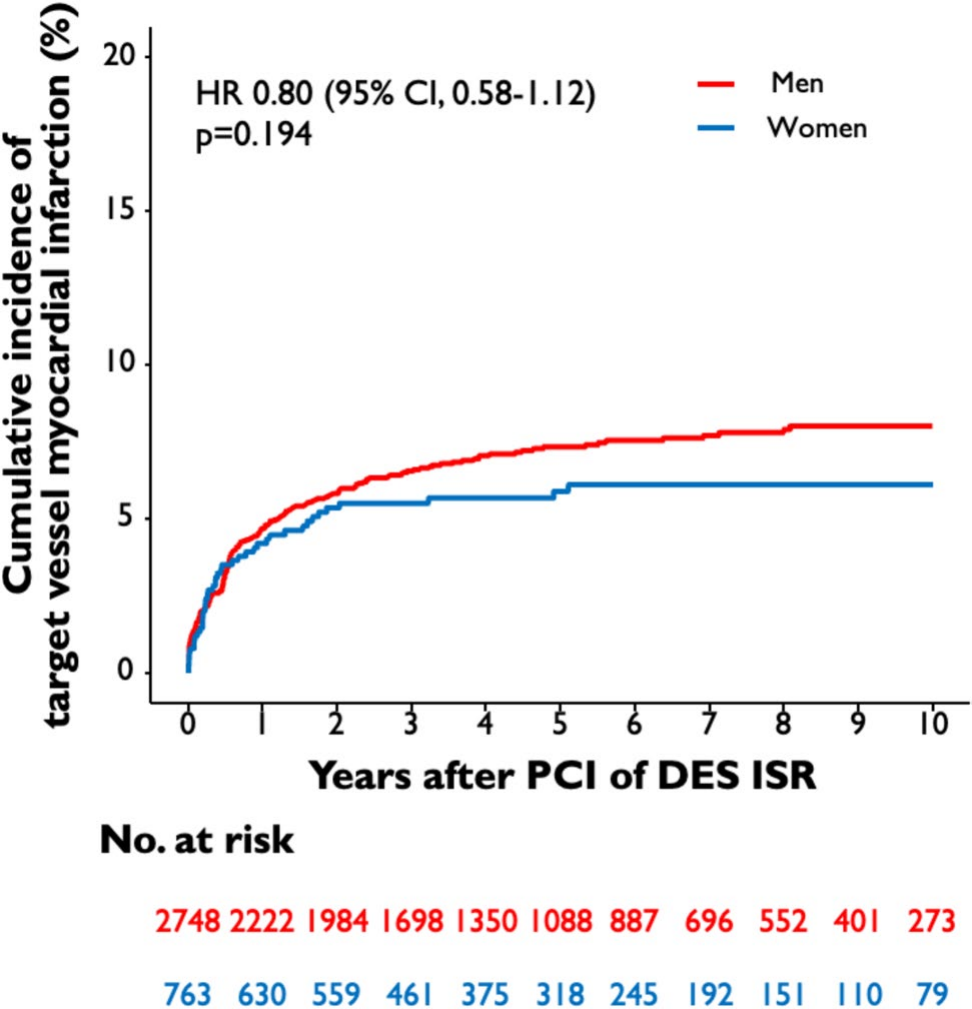
Sex-related 10-year cumulative incidence of TVMI. The figure demonstrates the cumulative incidence function curve and HR with accompanying 95% CI. CI, confidence interval; DES, drug-eluting stent; HR, hazard ratio; ISR, in-stent restenosis; PCI, percutaneous coronary intervention; TLR, target lesion revascularization; TVMI, target vessel myocardial infarction

The median follow-up duration was 5.9 years (75% confidence interval [CI] 3.5; 9.4 years). 19% of the total patient cohort (670 of 3,511 patients) had an incomplete follow-up of 9 years or less. Ten-year all-cause mortality after PCI of DES-ISR was significantly higher in female than in male patients (54.7% vs. 46.0%; HR 1.18 [95% CI 1.02–1.35]; *p* = 0.022) **(**Fig. 1**)**. A total of 27.6% of female patients and 31.7% of male patients underwent TLR after 10 years (HR 0.89 [95% CI 0.76–1.04]; *p* = 0.154) **(**Fig. 2**),** TVMI rate was 6.1% in female and 8.0% in male patients (HR 0.80 [95% CI 0.58–1.12]; *p* = 0.194) (Fig. 3). At 10-year follow-up TVR rate was 20.8% in female and 28.5% in male patients (HR 0.73 [95% CI 0.61–0.87]; *p* < 0.001), NTVR rate was 31.1% in female and 41.3% in male patients (HR 0.74 [95% CI 0.64–0.86]; *p* < 0.001). NTVMI rate was 6.2% in female and 7.9% in male patients (HR 0.77 [95% CI 0.55–1.10]; *p* = 0.148). In 1.7% of the female patients and 1.9% of the male patients, ST was detected after 10 years (HR 0.85 [95% CI 0.46–1.61]; *p* = 0.626). An overview of the clinical outcomes is presented in Table 3.

**Table 3:** Clinical outcomes

	Ten-year event rate of female patients (*n* = 763)	Ten-year event rate of male patients (*n* = 2748)	HR_unadj_ (95% CI)	*p* value
All-cause mortality	54.7	46.0	1.18 (1.02–1.35)	0.022
Target lesion revascularization	27.6	31.7	0.89 (0.76–1.04)	0.154
Target vessel myocardial infarction	6.1	8.0	0.80 (0.58–1.12)	0.194
Non-target vessel myocardial infarction	6.2	7.9	0.77 (0.55–1.10)	0.148
Target vessel revascularization	20.8	28.5	0.73 (0.61–0.87)	< 0.001
Non-target vessel revascularization	31.1	41.3	0.74 (0.64–0.86)	< 0.001
Stent thrombosis	1.7	1.9	0.85 (0.46–1.61)	0.626

Data shown as Kaplan–Meier event rates for all-cause mortality and cumulative incidences after accounting for the competing risk of death for the other outcomes. Data shown as percentages

Target vessel revascularization is defined as revascularization in the target vessel outside the target lesion

HR, hazard ratio

### Multivariable analysis

According to the multivariable Cox proportional hazards model, age [for each 1-year increase, HR_adj_ 1.06; (95% CI: 1.06–1.07); *p* < 0.001], diabetes mellitus [HR_adj_ 1.51; (95% CI: 1.33–1.71); *p* < 0.001], clinical presentation with ACS [HR_adj_ 1.52; (95% CI: 1.34–1.73); *p* < 0.001], prior MI [HR_adj_ 1.19; (95% CI: 1.05–1.34); *p* = 0.007], higher creatinine on admission [HR_adj_ 1.42; (95% CI: 1.29–1.56); *p* < 0.001] and higher CRP on admission [HR_adj_ 1.01; (95% CI: 1.00–1.01); *p* < 0.001] were independently associated with higher likelihood of all-cause mortality after 10 years. Higher ejection fraction [HR_adj_ 0.98; (95% CI: 0.98–0.99); *p* < 0.001], prescription of P2Y12 inhibitor on discharge [HR_adj_ 0.71; (95% CI: 0.52–0.97); *p* = 0.031] and prescription of statine on discharge [HR_adj_ 0.63; (95% CI: 0.51–0.79); *p* < 0.001] were found out to be independent preventive parameters of all-cause mortality after 10 years.

Diabetes mellitus [HR_adj_ 1.20; (95% CI: 1.02–1.42); *p* = 0.027] and the use of DCB [HR_adj_ 1.30; (95% CI: 1.05–1.61); *p* = 0.016] were independently associated with higher likelihood of TVR after 10 years. Female sex [HR_adj_ 0.75; (95% CI: 0.60–0.93); *p* = 0.008] and age [for each 1-year increase, [HR_adj_ 0.99; (95% CI: 0.98–1.00); *p* = 0.004] were found out to be independent preventive parameters of TVR after 10 years.

Diabetes mellitus [HR_adj_ 1.28; (95% CI: 1.13–1.46); *p* < 0.001], clinical presentation with ACS [HR_adj_ 1.32; (95% CI: 1.16–1.51); *p* < 0.001] and multivessel CAD [HR_adj_ 3.91; (95% CI: 2.80–5.45); *p* < 0.001] were independently associated with higher likelihood of NTVR after 10 years. Female sex [HR_adj_ 0.79; (95% CI: 0.67–0.93); *p* = 0.004] and higher creatinine on admission [HR_adj_ 0.82; (95% CI: 0.68–0.98); *p* = 0.034] were found out to be an independent preventive parameter of NTVR after 10 years. A summary of multivariable-adjusted analyses is presented in Table 4.

**Table 4:** Multivariable analysis

All-cause mortality	HR_adj_ (95% CI)	*p* value
Sex	0.98 (0.85–1.14)	0.827
Age	1.06 (1.06–1.07)	< 0.001
Diabetes mellitus	1.51 (1.33–1.71)	< 0.001
Clinical presentation with ACS	1.52 (1.34–1.73)	< 0.001
Multivessel CAD	1.20 (0.94–1.53)	0.142
Prior MI	1.19 (1.05–1.34)	0.007
Prior CABG	1.07 (0.961–1.26)	0.421
Ejection fraction	0.98 (0.98–0.99)	< 0.001
Creatinine on admission	1.42 (1.29–1.56)	< 0.001
CRP on admission	1.01 (1.00–1.01)	< 0.001
ASA on discharge	0.96 (0.67–1.38)	0.834
P2Y12 inhibitor on discharge	0.71 (0.52–0.97)	0.031
Statine on discharge	0.63 (0.51–0.79)	< 0.001
Drug-coated balloon	1.02 (0.83–1.25)	0.871

Target vessel revascularization	HR_adj_ (95% CI)	*p* value
Sex	0.75 (0.60–0.93)	0.008
Age	0.99 (0.98–1.00)	0.004
Diabetes mellitus	1.20 (1.02–1.42)	0.027
Clinical presentation with ACS	1.17 (0.99–1.39)	0.071
Multivessel CAD	1.14 (0.87–1.49)	0.337
Prior MI	1.01 (0.86–1.18)	0.928
Prior CABG	1.18 (0.95–1.48)	0.129
Ejection fraction	1.00 (1.00–1.01)	0.5
Creatinine on admission	0.84 (0.67–1.07)	0.16
CRP on admission	1.00 (0.99–1.01)	0.634
ASA on discharge	1.22 (0.62–2.39)	0.56
P2Y12 inhibitor on discharge	1.34 (0.78–2.31)	0.292
Statine on discharge	0.81 (0.59–1.12)	0.207
Drug-coated balloon	1.30 (1.05–1.61)	0.016

Non-target vessel revascularization	HR_adj_ (95% CI)	*p* value
Sex	0.79 (0.67–0.93)	0.004
Age	0.99 (0.99–1.00)	0.088
Diabetes mellitus	1.28 (1.13–1.46)	< 0.001
Clinical presentation with ACS	1.32 (1.16–1.51)	< 0.001
Multivessel CAD	3.91 (2.80–5.45)	< 0.001
Prior MI	0.98 (0.86–1.11)	0.735
Prior CABG	1.14 (0.97–1.35)	0.119
Ejection fraction	1.00 (0.99–1.00)	0.403
Creatinine on admission	0.82 (0.68–0.98)	0.034
CRP on admission	1.00 (0.99–1.00)	0.153
ASA on discharge	0.92 (0.58–1.44)	0.706
P2Y12 inhibitor on discharge	1.26 (0.84–1.89)	0.257
Statine on discharge	0.83 (0.65–1.07)	0.148
Drug-coated balloon	1.17 (0.98–1.38)	0.077

ACS, acute coronary syndrome; ASA, acetylsalicylic acid; CABG, coronary artery bypass graft; CAD, coronary artery disease; CRP, C-reactive protein; HR_adj_, adjusted hazard ratio; MI, myocardial infarction

### Subgroup analysis

A subgroup analysis of the outcomes of interest (all-cause death, TVR and NTVR) through to 10 years after PCI of DES-ISR was performed for subgroups based on age (> 70 years versus ≤ 70 years), diabetes mellitus (yes versus no) and clinical presentation (acute coronary syndrome versus chronic coronary syndrome) without revealing significant interactions. An overview of the subgroup analysis is presented in Table 5.

**Table 5:** Subgroup analysis

Sex-related all-cause mortality	HR (95% CI)	*P*_int_ value
Age		
> 70 years	0.91 (0.76–1.07)	0.662
≤ 70 years	1.16 (0.88–1.55)	
Diabetes mellitus		
Yes	0.98 (0.78–1.23)	0.088
No	1.00 (0.83–1.21)	
Clinical presentation		
ACS	1.02 (0.80–1.30)	0.863
CCS	0.98 (0.81–1.17)	

Sex-related target vessel revascularization	HR (95% CI)	*P*_int_ value
Age		
> 70 years	0.66 (0.49–0.89)	0.176
≤ 70 years	0.87 (0.64–1.17)	
Diabetes mellitus		
Yes	0.55 (0.38–0.80)	0.055
No	0.90 (0.69–1.17)	
Clinical presentation		
ACS	1.00 (0.69–1.44)	0.055
CCS	0.65 (0.50–0.85)	

Sex-related non-target vessel revascularization	HR (95% CI)	*P*_int_ value
Age		
> 70 years	0.77 (0.62–0.96)	0.546
≤ 70 years	0.81 (0.63–1.04)	
Diabetes mellitus		
Yes	0.78 (0.61–1.01)	0.9
No	0.79 (0.63–0.98)	
Clinical presentation		
ACS	0.78 (0.58–1.05)	0.724
CCS	0.80 (0.66–0.97)	

ACS includes patients with unstable angina, NSTEMI, STEMI; CCS includes patients with stable angina

ACS, acute coronary syndrome; CCS, chronic coronary syndrome; CI, confidence interval; HR, hazard ratio; *P*_int_, *p* value for interaction

### Recurrent ISR

In female patients, 1124 lesions were treated for initial ISR, and in male patients, 4373 lesions. In these female and male patients, 268 and 1.096 lesions, respectively, were treated for a second recurrent ISR with DES implantation in 40.7% in female and 42.8% in male patients. 95 lesions in female and 416 lesions in male patients were treated for third recurrent ISR with DES implantation in 30.5% in female and 33.9% in male patients. Moreover, 50 lesions in female and 207 lesions in male patients were treated for fourth recurrent ISR with DES implantation in 28.0% in female and 31.9% in male patients. In both groups, recurrent ISR were significantly less likely treated with DES implantation in comparison to first ISR (*p* < 0.001; *p* < 0.001). No excess risk for treatment of recurrent ISR was associated with female sex [HR 0.94 (95% CI: 0.81–1.10), HR_adj_ 1.02; (95% CI: 0.88–1.18)].

## Discussion

This analysis addresses the lack of data concerning sex-related differences in long-term outcomes after PCI of DES-ISR. The major findings are:

- After age adjustment, 10-year all-cause mortality after PCI of DES-ISR did not differ significantly between female and male patients.
- At 10-year follow-up, the risk of TVR and NTVR was lower in female than in male patients.

Based on a report of the American Heart Association, young female patients have a lower risk of cardiovascular disease compared to young male patients; at age 60–79 years, female catch up with male patients and exceed the male patient’s risk at age 80 [18, 19]. Likewise, the risk factor profile is somewhat different [6, 14, 20]. In agreement with this literature, at baseline, several clinical characteristics were different between female and male patients treated for ISR and studied here, which requested an adjusted comparison. In our analysis, female patients were significantly older and suffered more often from diabetes. Likewise, the baseline characteristics of the DECADE cooperation (Adverse Events and Coronary Artery Disease Progression)—which analyzed sex-related outcomes of five randomized controlled PCI trials with DES implantation for native coronary artery stenosis—showed that female were older than male patients, present more frequently with diabetes, whereas a higher proportion of male patients present as current smokers, with multivessel CAD and with prior CABG surgery or prior MI [6]. Despite these findings regarding native CAD, an angiographic follow-up study including of 4374 demonstrated that ISR occurred less frequently in female than in male patients—although higher rates of diabetes and smaller vessel size were found in female patients, which represent typical risk factors for the occurrence of ISR [20]. While male patients predominantly present with focal stenosis of the coronary artery tree, female patients more likely suffer from diffuse CAD involving epicardial vessels [6, 21]. Smaller vessel size and higher rates of microvascular disease may underestimate the occurrence of relevant CAD in female patients [6, 22]. Taking these observations into account, the current perception is that male patients seem to have a higher risk for native CAD as well as for ISR, that does not depend on classical cardiovascular risk factors.

The DECADE cooperation found higher all-cause mortality rates in female patients; however, after adjustment for age, the differences in all-cause mortality have leveled out [6]. This result is consistent with data analyzed in the current study where 10-year all-cause mortality rate in female patients was significantly higher than in male patients, but at presentation, female were 3.7 years older than male patients. The multivariable adjustment revealed age to be the main confounder and demonstrated that age-adjusted sex-related all-cause mortality rates did not significantly differ in the long term. Moreover, the DECADE cooperation found out female sex to go along with a lower risk of revascularization of the target lesion, target vessel and non-target vessel at 10-year follow-up [6]. These results are in line with data of the current analysis, which confirmed higher rates of TVR and NTVR in male patients being treated for DES-ISR, long-term TLR rates were found to be numerically lower in female than in male patients without being statistically significant. In addition, a meta-analysis including 21 randomized PCI trials and 32,877 patients being treated for native coronary artery stenosis supports the present findings, since significantly higher rates of all-cause death, cardiovascular death and major adverse cardiovascular events after 5 years of follow-up were found in female patients [14].

However, another multicenter cohort study evaluating sex-related outcomes in older patients (≥ 75 years) with non-ST-elevation myocardial infarction (NSTEMI) did not yield relevant differences in 5-year follow-up between female and male patients [8]. On the contrary, in young patients (≤ 55 years) with acute coronary syndrome, sex-related differences in clinical outcomes become more evident with female patients presenting a higher risk population with more comorbidities and cardiovascular risk factors in comparison to male patients [23]. These data are consistent with the results of a multicenter registry analyzing outcomes of 10,963 patients after PCI, where the subgroup of young female patients (< 50 years) presented increased rates of target vessel and target lesion failure based on an angiographic less severe CAD in comparison to male patients of the same age; however, outcomes in older patients did not yield sex-specific differences [24].

In addition to inherent biological factors, sex-specific differences in long-term outcomes may be influenced by a more atypical symptom manifestation in female patients [25] which may result in lower rates of repeat invasive angiography due to unrecognized or misdiagnosed symptoms [6, 26]. Moreover, female patients tend to present more likely with plaque erosions potentially being undetected in the invasive angiography [27]. Lower rates of repeat revascularization in female patients have been reported previously [28, 29] although similar recommendations are being made in the guidelines [30]. Lower rates of revascularization therapy in female than in male patients might be a potential reason for increased clinical event rates in young female patients with acute coronary syndrome [31]. Moreover, higher mortality rates in female patients after PCI are mainly caused by an older age at diagnosis of CAD but may also be attributed to a more extensive cardiovascular risk profile compared to male patients [32]. Besides, lower revascularization rates in female patients in the long term might among others be caused by higher mortality rates in female patients reducing the potential of next revascularization.

## Limitations

Several limitations apply to our analysis. First, it is an observational retrospective study and so subject to all limitations of this design. Thus, some information is not available for every patient, e.g., exact cause of death or selected procedural characteristics (like the initial DES-type) of patients who were initially treated elsewhere. Second, substantial advances in interventional treatment and anti-thrombotic therapy were made during the inclusion period. This may be a reason why DCB was used only in the minority of the patients. In addition, intravascular imaging has not been systematically applied. Furthermore, the median follow-up duration was 5.9 years (75% confidence interval [CI] 3.5; 9.4 years) with 19% of the total patient cohort (670 of 3511 patients) having an incomplete follow-up of 9 years or less.

## Conclusions

In the long-term patients undergoing PCI of DES-ISR show high rates of adverse events, which seem comparable to native CAD. In this patient cohort, age-adjusted 10-year all-cause mortality did not differ significantly between female and male patients. There was a signal for a lower risk of TVR and NTVR in female patients even after age adjustment. Further trials with increased involvement of female patients are required to investigate baseline-adjusted sex-related differences in long-term outcomes after PCI of DES-ISR as well as optimal treatment strategies for both, female and male patients.

## Data Availability

The data that support the findings of this study are available from the ISAResearch-Center, Munich, Germany*.* The authors state no funding involved. The study was approved by the local ethics board of the Technical University Munich, Germany and adheres to the Declaration of Helsinki.

## Abbreviations

ACS: Acute coronary syndrome
BA: Balloon angioplasty
CABG: Coronary artery bypass graft
CAD: Coronary artery disease
CI: Confidence interval
DCB: Drug-coated balloon
DES: Drug-eluting stent
HR: Hazard ratio
ISR: In-stent restenosis
MI: Myocardial infarction
NSTEMI: Non-ST-elevation myocardial infarction
NTVMI: Non-target vessel myocardial infarction
NTVR: Non-target vessel revascularization
PCI: Percutaneous coronary intervention
ST: Stent thrombosis
TLR: Target lesion revascularization
TVMI: Target vessel myocardial infarction
TVR: Target vessel revascularization
